# Flocculation of Cellulose Microfiber and Nanofiber Induced by Chitosan–Xylan Complexes

**DOI:** 10.3390/nano13172420

**Published:** 2023-08-25

**Authors:** Gabriela Adriana Bastida, Quim Tarrés, Roberto Aguado, Marc Delgado-Aguilar, Miguel Ángel Zanuttini, María Verónica Galván

**Affiliations:** 1 Institute of Cellulosic Technology, Faculty of Chemical Engineering (FIQ-CONICET), National University of the Litoral, Santiago del Estero 2654, Santa Fe S3000AOJ, Argentina; gabriela.bastida@udg.edu (G.A.B.); mzanutti@fiq.unl.edu.ar (M.Á.Z.); vgalvan@fiq.unl.edu.ar (M.V.G.); 2 LEPAMAP-PRODIS Research Group, University of Girona, Maria Aurèlia Capmany 61, 17003 Girona, Spain; roberto.aguado@udg.edu (R.A.); m.delgado@udg.edu (M.D.-A.)

**Keywords:** flocculation, xylan, chitosan, nanocellulose, gel point, viscosity, floc size

## Abstract

This study aims to provide a comprehensive understanding of the key factors influencing the rheological behavior and the mechanisms of natural polyelectrolyte complexes (PECs) as flocculation agents for cellulose microfibers (CMFs) and nanofibers (CNFs). PECs were formed by combining two polyelectrolytes: xylan (Xyl) and chitosan (Ch), at different Xyl/Ch mass ratios: 60/40, 70/30, and 80/20. First, Xyl, Ch, and PEC solutions were characterized by measuring viscosity, critical concentration (c*), rheological parameter, ζ-potential, and hydrodynamic size. Then, the flocculation mechanisms of CMF and CNF suspensions with PECs under dynamic conditions were studied by measuring viscosity, while the flocculation under static conditions was examined through gel point measurements, floc average size determination, and ζ-potential analysis. The findings reveal that PEC solutions formed with a lower xylan mass ratio showed higher intrinsic viscosity, higher hydrodynamic size, higher z-potential, and a lower c*. This is due to the high molecular weight, charge, and gel-forming ability. All the analyzed solutions behave as a typical non-Newtonian shear-thinning fluid. The flocculation mechanisms under dynamic conditions showed that a very low dosage of PEC (between 2 and 6 mg PEC/g of fiber) was sufficient to produce flocculation. Under dynamic conditions, an increase in viscosity indicates flocculation at this low PEC dosage. Finally, under static conditions, maximum floc sizes were observed at the same PEC dosage where minimum gel points were reached. Higher PEC doses were required for CNF suspensions than for CMF suspensions.

## 1. Introduction

In many industrial processes, such as paper manufacturing, water treatment, or minerals processing, solid–liquid separation plays a crucial role. Flocculating agents are commonly employed for this purpose, with water-soluble polymers being widely used due to their influence on both flocculation mechanisms and separation outcomes [1]. 

The use of nanocellulose as a reinforcing agent in papermaking has gained significant traction due to its appealing properties, such as high strength, excellent stiffness, and a large surface area [2,3]. In addition, due to its interesting characteristics, it can also be used in membranes [4], electronic devices [5], biomedical applications [6], and bio-composites [7]. The suspensions of cellulose microfibers (CMF) or cellulose nanofibers (CNF) are differentiated based on their characteristic size [8]. According to the Technical Association of Pulp and Paper Industry (TAPPI-WI-30212010), CNFs can be considered as a chain of cellulose molecules with a high aspect ratio (length–diameter), with a diameter of 5 to 30 nm and length of 10 times or more than its diameter. On the other hand, CMFs are a type of cellulose-structured material containing multiple elementary fibrils with both widths of 10 to 100 nm and lengths of 0.5 to 10 mm. 

A challenge in materials produced from CNF/CMF suspensions is the lengthy preparation time due to drainage difficulties. To expedite drainage, cationic polyelectrolytes have been used to control flocculation [9,10]. The agitation level and floc formation control after cationic polyelectrolyte addition are critical for achieving good sheet formation. However, there is an optimum flocculant dosage level beyond which flocculation rapidly deteriorates [11,12]. Alternatively, cationic polyelectrolyte complexes (PECs) offer another solution. These complexes are formed through electrostatic interactions and entropy gain between oppositely charged polyelectrolytes in dispersions. If they are properly formed, they can be advantageous for diverse applications, including films [13], coatings [14], biomedical uses [15], water purification [16], and as a strength additive for paper [17].

Our research group has previously analyzed the addition of micro/nanofibers (CMNF) in combination with PECs during paper formation [18]. PECs were based on natural polyelectrolytes such as chitosan (Ch) and xylan (Xyl). We found that these PEC-CMNF systems are a promising alternative because an excellent drainage capacity and excellent retention of fines and CNMF were obtained. Besides that, the mechanical properties of the final paper were notably improved, i.e., CMT, SCT, and internal delamination values were increased. The present paper continues and complements the analysis of the effect of these complexes on CMF and CNF suspensions. In this context, it is of interest to analyze the rheological behavior and interactions of our isolated system under conditions similar to those that are generally going to be used. That is, static or flowing at relatively high speeds, with turbulence and strong mechanical actions.

The major flocculation mechanisms involving polyelectrolytes and cellulosic fibers or CNFs are “charge neutralization” and “bridging” [19]. When PEC mechanisms are considered, a new situation arises as this leads to the formation of new particles. Nyström et al. [20] demonstrated enhanced flocculation in calcite dispersions using sodium polyacrylate–cationic starch complexes compared to individual polyelectrolytes alone, owing to the PECs’ larger, more rigid structure and better anchorage on particle surfaces.

Viscosity measurements can be used to analyze flocculation under dynamic conditions. The rheological properties of dilute CNF suspensions depend on the degree of aggregation of the nanofibrils, which gradually break down under shear, leading to a decrease in floc size [21]. 

In the dilute regime, electrostatic attraction or repulsion can alter the rheology, causing flocculation or dispersion [22]. While in concentrated conditions, the electrostatic attraction or repulsion causes changes in the strength of the interfibrillar interactions and in the value of the elastic limit, showing a viscoelastic behavior [23]. Other important properties in the case of fibers that can be considered rigid rods are the aspect ratio, particle density, and flexibility [24]. The rheological properties of PEC and CNF, as well as their combination, are important for their processing, transportation, storage, and pumping, but also due to their potential in several applications; for example, use as a rheology modifier in coatings and paints.

The flocculation of PEC-CNF suspensions under static conditions can be analyzed by determining the gel point. The gel point corresponds to the concentration of solids at which the primary flocs interconnect and form a self-supporting network [18] and is measured by sedimentation experiments for fibers [25], CMF [26], and CNF [27] suspensions. In colloidal suspension, Brownian motion, and attractive and repulsive forces (dispersion, electrostatic, and steric forces) affect the microstructure in the absence of flow [28]. This generates collisions, leading to fiber entanglement, and subsequent sedimentation [29]. However, the CNF's flocculation efficiency is highly dependent on its properties, such as electrical charges, size, and surface area. Furthermore, this parameter is significantly improved when chemically modified CNFs are used [30]. Studies of gel point have recently been carried out to evaluate CNF flocculation using polyelectrolytes [19,31,32]. However, to the best of our knowledge, this technique has not been used to assess CMF and CNF flocculation using PECs. Our study could contribute to important advances in this area.

Given these considerations, our study aims to investigate the viscosity properties and mechanisms involved in the enhanced flocculation induced by PECs based on chitosan and xylan. The rheological behaviors of polyelectrolytes, PECs, CMF, and CNF individually, as well as the PEC-CNF system, were analyzed. The dynamic and static floc properties were analyzed using viscosity measurements and sedimentation experiments, respectively.

One of the primary contributions of this study in the field of rheology lies in the thorough analysis of the mechanisms underlying the action of these complexes when employed as flocculants. This investigation uncovers a novel scenario that transcends the conventional realm of charge neutralization. These complexes defy classification as mere bridges or patches; instead, they catalyze the creation of entirely new particles. This intricate process involves a confluence of diverse effects, resulting in a combined action that significantly impacts the system.

Specifically, these flocculants trigger an augmentation of particle–particle interactions, thereby intensifying flow resistance even with minute additions. Moreover, the particle size distribution exhibits a bimodal nature due to the presence of composite micro- and nano-flocculants (CMFs), which comprise both macroscopic and nanoscale components. This dualistic composition introduces a complex interplay between the two size fractions, capable of both mitigating and amplifying effects. On one hand, the nanoscale entities operate as lubricants, showcasing their utility in reducing friction. Conversely, the microscale counterparts induce surface irregularities that foster heightened viscosity by bolstering the likelihood of particle–particle interactions.

## 2. Materials and Methods

### 2.1. Materials

Chitosan (Ch) was supplied by Sigma Aldrich (product number 448877, Sant Louis, MO, USA). The viscosity average molar mass was determined using a capillary viscometer Cannon-Fenske number 75 in a water bath at 25 °C, giving a value of MV: 190 kDa [33]. The degree of deacetylation (DD) of chitosan was determined using the linear potentiometric titration method reported by Jiang, Chen, and Zhong [34]. Briefly: Chitosan (0.2 g) was dissolved in 25 ml of 0.1 M HCl aqueous solution. The titrant used was a solution of 0.1 M NaOH. Under continuous stirring, titrant was added until the pH value of the solution reached a value of 2.0. From this point, the titration continued until the pH value of the solution reached a value of 6.0. Three replicates were performed for each simple. Determination resulted in a value of 79.6% ± 0.7% [33]. The chitosan was dissolved in diluted acetic acid to reach a concentration of 2.5 g/L, following the method described by Schnell et al. [35]. Xylan (Xyl) was obtained by alkaline extraction from sugar cane bagasse according to Solier et al. [36]. In brief, extractions were carried out at 50 °C and with a bagasse liquor ratio of 1:25, using an alkali charge of 40 % *w/w* on bagasse for 180 min. The hemicelluloses extracted were precipitated from the extraction liquor using ethanol at a 1:1 *v/v* liquor: ethanol ratio and were separated by centrifugation (15 min, 1800 G-force) after being reserved overnight at 4 °C. The composition: xylose 0.67, arabinose 0.18, glucuronic acid 0.02, insoluble lignin 0.07, and soluble lignin 0.06 *w/w*, and the weight-average molecular weight: 55 kDa were previously reported by Solier et al. [37].

### 2.2. Preparation and Characterization of Polyelectrolyte Complexes (PECs)

For the choice of the complexes, mass ratios are considered where they preserve their cationic charge and can act as flocculants with the anionic charges of the CMF and CNF suspensions. Therefore, PECs were formed at different mass ratios of Xyl/Ch: 60/40; 70/30, and 80/20, denoted as PEC_60/40_, PEC_70/30_, and PEC_80/20_, respectively. The PECs were formed from polyelectrolytes solution at maximum possible concentrations, close to their limits of solubility. For this, the xylan solution (10 g/L in water) was added to the chitosan solution (2.5 g/L chitosan in 2.5 g/L of acetic acid) under continuous stirring at 300 rpm and a flow rate of 90 mL/h. Before mixing, the pH values of both solutions were adjusted to 5.0. This pH was selected to maximize the electrostatic interaction between xylan and chitosan since both are similarly ionized under these conditions [35]. The particle sizes and ζ-potential of the cationic complex were determined at 25 °C, pH 5.0, and 0.1 wt% concentration using dynamic light scattering (DLS) with a Zetasizer Nano equipment (ZEN 3600). The refractive index used was 1.33, considering the high-water content of the PEC particles [33,38]. The viscosity of the medium used was 0.8937 mPa·s.

### 2.3. Preparation of CMFs and CNFs

CMFs and CNFs were derived from industrial bleached eucalyptus pulp (BEP) supplied by Suzano Papel e Celulose S.A. (Aracruz, Brazil) following the method outlined by Bastida et al. [27]. In brief, the pulp was soaked in water for 24 h and treated in a standard disintegrator (SCAN-C 18:65) for 5 min at 1.5% consistency. The resulting pulp suspension was centrifuged and stored. BEP fibers were mechanically pre-treated using a PFI mill at 10,000 revolutions and 10% of consistency in accordance with SCAN-C 18:65 standards. Then, 15 g of pulp was added to a reactor containing 750 mL oxalic acid considering two concentrations: 25 wt% and 50 wt%. The reaction was made at 90°C for 1 h under constant stirring at 250 rpm. After that, the solution was filtered, and the pulp was washed until the conductivity of the filtrate was reduced to 20 µS/cm. Finally, the pulp was neutralized to pH 7.0 with NaOH solution. The pulp was fibrillated by five passes at 0.75 wt% using a pilot-scale pressurized homogenizer (SIMES S.A, Santa Fe) at 300 bar. Table 1 shows the characteristics of the fibrillated cellulose obtained. Two types of micro/nanofibers are observed. One (named here as CMF) corresponds to the 25% oxalic acid treatment showing a low nanofibrillation yield and the other (named here as CNF) corresponds to 50% oxalic acid treatment that presents a higher nanofibrillation yield.

The nanofibrillation yield was determined by centrifuging (2800 G-force) an aqueous suspension of 0.1 wt% CMF and CNF for 20 min. The dry weight of the supernatant was obtained from the difference between the initial weight (*W_i_*) and the centrifugation sediment (*W_f_*) according to the following to equation:

$$Yield\;\left(\%\right)=\left[\frac{W_{i}-W_{f}}{W_{i}}\right]\times 100$$

### 2.4. Individual Rheological Behavior of Polyelectrolytes and PECs

Before all viscosity determinations, polyelectrolytes, and PECs solutions were sonicated for 1 min using a Sonics & Materials ultrasonic homogenizer (500 W, 40% amplitude) to ensure their adequate dispersion.

#### 2.4.1. Determination of the Critical Concentration (c*) and the Intrinsic Viscosity [η]

The dynamic viscosity of polyelectrolytes solutions (Xyl and Ch) and PEC suspensions with different mass ratios was determined using a Brookfield viscometer LVT with an Ultra-Low Adapter (ULA) spindle similar to the method proposed by Albornoz-Palma et al. [39] and Bastida et al. [27]. The configuration of ULA corresponds to a double-cylinder geometry (cup radius: 13.7 mm; spindle radius: 12.5 mm). In brief, viscosity measurements were made at different concentrations (from 0.01 wt% to 0.5 wt%) at a shear rate of 73.38 1/s. In all cases, samples were conditioned in a thermostated bath at 25 °C for 1 h, and then, they were shaken for 1 min in a vortex immediately before measurement. Then, the critical concentration (c*) of suspensions was obtained from the change in the slope of specific viscosity (*η_sp_*) as a function of polyelectrolytes or PEC concentration. The *η_sp_* is calculated with the following equation:

$$\eta_{sp}=\frac{\eta -\eta_{0}}{\eta_{0}}$$

where *η* and *η*_0_ are the viscosity of the solution and solvent, respectively.

The intrinsic viscosities, [*η*] (mL/g), can be obtained in the dilute region (Newtonian region) extrapolating the linear fit to a zero concentration according to the following equation:

$$\left[\eta \right]={lim}_{\to 0}\frac{\eta_{sp}}{c}={lim}_{\to 0}\eta_{red}$$

where *c* is the concentration of the dispersion (g/mL), *η_red_* is the reduced viscosity (mL/g).

#### 2.4.2. Rheological Parameters

Solutions of xylan, chitosan, and PECs were prepared at concentration: 0.3 wt%. The observed apparent viscosity (*η_app_*) was measured at 25 °C and considering shear rates (*γ*) of 7.34, 14.68, 36.69, and 73.38 1/s corresponding to a rotational speed of 6, 12, 30, and 60 rpm. The *η_app_* was calculated from the following equation:

$$\eta_{app}=\frac{shear\;stress\;(t)}{shear\;rate\;\left(\dot{\gamma }\right)}\times 100$$

τ=z·α

where *z* is the cylinder constant and *α* is the instrument reading.

### 2.5. Flocculation of PEC-CMF and PEC-CNF Systems at Dynamic Conditions

#### Viscosity

To simulate the effect of PEC-CMF and PEC-CNF interactions at dynamic conditions, the rheology was studied using a Brookfield viscometer LVT at a shear rate 73.38 1/s.

### 2.6. Flocculation of PEC-CMF and PEC-CNF Systems under Static Conditions

#### 2.6.1. Gel Point

The gel point was determined using an adaptation of the sedimentation method proposed by Raj et al. [31] for microfibrillated cellulose–polyelectrolyte suspensions. In brief: experiments were conducted at initial solids concentrations (C_0_) of CMF or CNF from 0.02 wt% to 0.06 wt%. In addition, solutions of 0.25 mg/mL of PECs were prepared. Then, different dosages were added to CMF or CNF suspensions (from 1 to 20 mg PEC/g fiber). The suspension was stirred for 2 min, and then poured into graduated cylinders to reach an original suspension height (h_0_). After 48 h, the height of sediment in the cylinder (h_s_) was measured. Initial solid concentration (C_0_) versus the ratio of sediment height (h_s_) to initial suspension height (h_0_) was plotted and fitted to a quadratic equation. The linear term of the fit was taken as the gel point (Supplementary Material Tables S1 and S2). Finally, the gel point is plotted as a function of the dosage added (mg PEC/g of CMF or CNF).

#### 2.6.2. Average Flocs Size

The size distributions of CMF and CNF flocs were obtained by direct photographic images and optical microscopy (Leica Microsystems Instrument), respectively. CMF and CNF suspensions were diluted to 0.02 wt% and different dosages of PECs were added. The suspensions were added in a petri dish and a few drops of a Congo Red dye were added to improve visualization. A number of 450 size measurements were performed using Image J processing software (version 1.41).

#### 2.6.3. Zeta Potential

The ζ-potential measurements were performed using a Zetasizer Nano Series (Malvern Panalytical, Malvern, UK) at 25 °C. Different dosages of PEC were added at 0.05 wt% CMF or CNF suspension and stirred for 2 min. Then, the suspension was centrifuged at 1800 G-force for 20 min to remove big aggregates and retain colloids. The supernatant containing colloidal nanocellulose was used to measure the ζ-potential [31].

## 3. Results

### 3.1. Individual Rheological Behavior of Polyelectrolytes and PECs

#### 3.1.1. Determination of Critical Concentration (c*) and the Intrinsic Viscosity [η]

**Figure 1:**
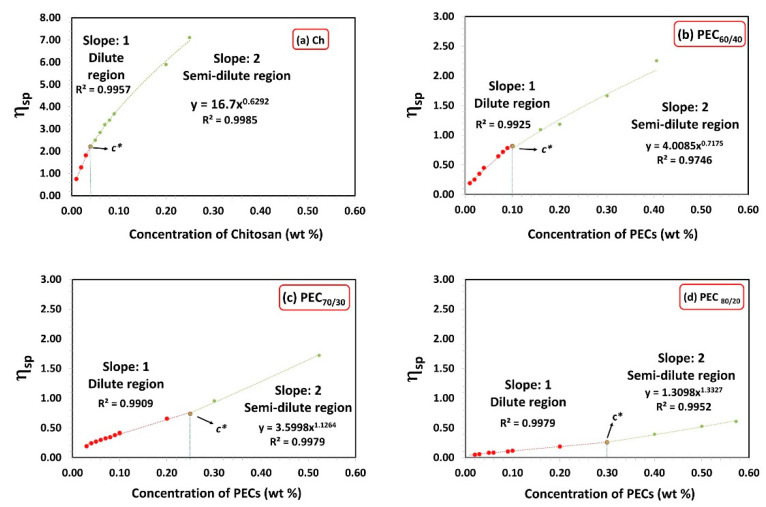
Specific viscosity as a function of concentration of chitosan and PECs with different mass ratios Xyl/Ch: 60/40, 70/30, and 80/20 (PEC_60/40_, PEC_70/30,_ and PEC_80/20_). Shear rate: 73.38 1/s.

Figure 1 illustrates the specific viscosity (*η_sp_*) as a function of chitosan and PECs concentration for different mass ratios (PEC_60/40_, PEC_70/30_, and PEC_80/20_). The critical concentration defines the transition point from the dilute region to the semi-dilute region, where molecular interactions increase, and interpenetration occurs [40,41]. In the concentration range above c* and below the critical entanglement concentration (c_e_), polymer chains can overlap effectively without becoming entangled [42]. Table 2 presents the c* values for Xyl, Ch, PEC solutions, as well as CMF and CNF suspensions. Chitosan and xylan solutions exhibit low and high c* values, respectively. The c* value is influenced by the molecular size and conformation of the polymer, higher molecular weight, and more rigid conformation, owing to higher charge, leading to lower c* [43].

It is important to note that in the semi-diluted regime (c > c*), the zero-shear viscosity of Ch is proportional to the concentration (h_0_ ~ c^0.63^). The exponent is slightly greater than 0.5, which is the value proposed by the empirical Fuoss law (h_0_ ~ c^0.5^) for polyelectrolyte solutions [44].

PECs display intermediate c* values between Xyl and Ch. As expected, increasing the amount of Ch in the PEC decreases its critical concentration.

On the other hand, CMF and CNF have lower c* values compared to those of PECs. This can be attributed to their ability to form entangled network-like structures, leading to highly viscous suspensions even at low concentrations, mainly due to the high aspect ratio and hydrophilicity of nanocellulose as indicated by Tingaut et al. [45].

Particularly, CMF has a lower c* than CNF, due to its larger average size (Table 2) and length (Table 1). However, CMF has a lower surface charge than CNF, suggesting that the morphology of micro/nanofibrils has a greater influence on c* than their charges.

**Figure 2:**
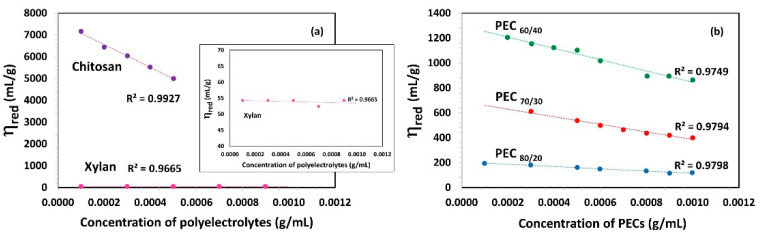
Reduced viscosity (*η_red_*) as a function of concentration of (**a**) polyelectrolytes solutions (Xylan and Chitosan) and (**b**) complexes (PECs) with different mass ratio Xyl/Ch: 60/40, 70/30, and 80/20 (PEC_60/40_, PEC_70/30_, and PEC_80/20_). Shear rate: 73.38 1/s; temperature: 25 °C.

Figure 2a shows the relationship between reduced viscosity (*η_red_*) and the concentration of polyelectrolytes. In the case of chitosan, its viscosity decreases linearly as the concentration increases. Gartnet and López [46] observed that the chitosan solution, when free from salts, exhibits a high intrinsic viscosity due to repulsions between the charged chains, leading to increased rigidity. In contrast, the dynamic viscosity of the Xyl solution is found to be very low, similar to that of water, with no significant changes observed as the concentration increases.

Figure 2b presents the viscosity (*η_red_*) as a function of PEC concentration and indicates that an increase in the Xyl mass ratio in PEC results in a decrease in *η_red_*. This suggests that the viscosity of PEC solution decreases as the PEC size decreases. A lower size implies the lower possibility of an interaction between PECs. With an increasing Xyl mass ratio in PEC, viscosity is also reduced as a consequence of a lower electrical charge and thus, a decrease in electrostatic repulsion forces between them. Notably, the PEC solution formed with the highest proportion of chitosan (PEC_60/40_) exhibits the highest viscosity among the studied mixtures.

The intrinsic viscosity [η] values were determined in the dilute region (c ˂ c*) through extrapolation of the linear fit to zero concentration, as depicted in Figure 2. These [*η*] values are summarized in Table 2. Chitosan demonstrated an exceptionally high intrinsic viscosity [*η*] value of 7610.0 mL/g, which is consistent with its substantial molecular weight, charge, and gel-forming ability. In addition, Kwang and Shin, attribute the high intrinsic viscosity of chitosan, compared to other biopolymers with similar molecular weight, to the rigidity of the b-(1,4) glucose linkages of cellulose that make up its structure [43]. On the other hand, the intrinsic viscosity values of xylan aligned with the order of magnitude previously reported by Silva et al. [47]. Specifically, two xylan fractions, termed xylan A and xylan B, were isolated from corn cobs using three different processes, and their intrinsic viscosity values were found to be 56 and 75 mL/g, respectively. As the authors also suggested, the observed differences in viscosity values can be attributed to the polymers’ distinct hydrodynamic volumes and inherent nature.

#### 3.1.2. Rheological Parameters

**Figure 3:**
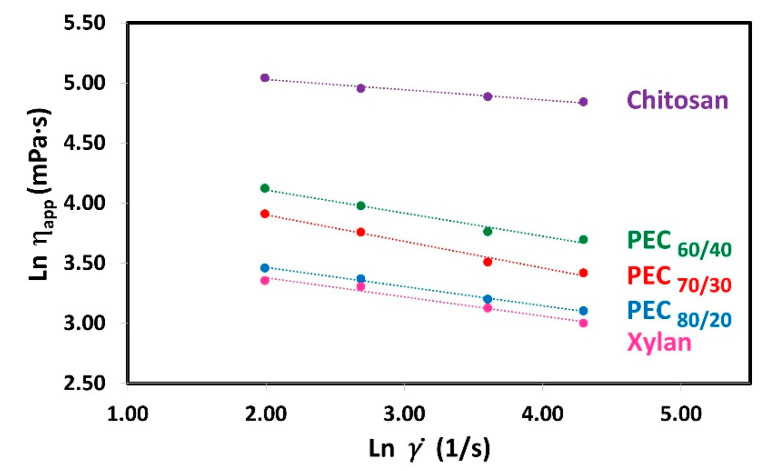
Plots of ln(*η_app_*) versus ln($\dot{\gamma }$) for shear rate (7.34, 14.68, 36.69, and 73.38 1/s) for aqueous solutions of Xyl and Ch, and their complexes (PECs) at different mass ratio Xyl/Ch: 60/40, 70/30, and 80/20 (PEC_60/40_, PEC_70/30_, and PEC_80/20_). Temperature: 25 °C; concentration: 0.3 wt%.

To know which is the rheological behavior of polyelectrolyte solutions (Xyl and Ch) and complexes suspension, the relationships between the shear rate ($\dot{\gamma }$) and the apparent viscosity (*η_app_*) were analyzed in an ln-ln graph for Xyl, Ch, and PEC solutions at 0.3% weight concentration as shown in Figure 3. Polymer solutions when subjected to shear stress can have different types of rheological behaviors: Newtonian, Pseudoplastic, or Dilatant. Newtonian fluids are those that keep their apparent viscosity constant as the shear rate increases. On the other hand, fluids that decrease or increase their apparent viscosity with increasing shear rate are called pseudoplastic or dilatant, respectively. The data show a clear decrease in apparent viscosity as the shear rate increases, indicating their pseudoplastic behavior [48,49]. Moreover, the plot reveals straight lines, suggesting that the Power Law model (also known as the Ostwald model) is suitable for representing the data within this range of shear rate:

$$\eta =K{\dot{\gamma }}^{n-1}$$

where *K* is the consistency index and *n* is the flow behavior index. Figure 3 shows the linear regression of the Power Law Model. Where the slope gives the value of *n*^−1^ and the y-intercept gives the value of *K*. The rheological parameters obtained in this study are presented in Table 3.

Additionally, in the Supplementary Material the plots of *η_app_* versus shear rate (7.34; 14.68; 36.69, and 73.38 1/s) and different concentrations for PECs, at 25 °C, are shown (Supplementary materials, Figure S1).

The rheological parameters obtained in this study are presented in Table 3. Notably, the *K* value for xylan is the lowest, while for chitosan, it is the highest. As the mass ratio of chitosan in the PEC increases, the value of *K* also increases, suggesting a rise in apparent viscosity. Previous studies have demonstrated that these PECs exhibit a spherical shape [33]. Considering the semi-rigid nature and persistence length of both polyelectrolytes’ chains [50,51], the PECs can be modeled as charge-neutralized cores surrounded by stabilizing shells as was suggested by others [52]. As the amount of xylan increases, the space between polymer chains decreases, resulting in a more compact complex. This compact structure may explain the reduction in size, decrease in charges, and lower viscosity.

Furthermore, with an increase in the mass ratio of chitosan in the PEC, the value of *K* rises, indicating a higher apparent viscosity. Moreover, as expected, when the concentration of polyelectrolytes and PEC increases, the value of *K* also increases (Supplementary materials, Table S3). This effect is attributed to the closer proximity of charges, leading to stronger repulsive forces between the chains. Consequently, the resistance of the chains to movement increases, resulting in the observed increase in viscosity, which has been described in a similar polyelectrolyte system by Wyatt et al. [53].

When the Power Law presents a flow behavior index (*n*) equal to 1, there is a Newtonian behavior. If *n* is lower or greater than 1.0, there is a pseudoplastic and dilatant behavior, respectively. Both polyelectrolytes and the PECs exhibit n values less than 1.0 (ranging from 0.779 to 0.915), indicating a non-Newtonian pseudoplastic behavior. In polymer solutions, shear thinning occurs due to the disentanglement of polymer chains during flow. At rest, high molecular weight polymers are entangled and randomly oriented. However, under agitation at a sufficiently high rate, these highly anisotropic polymer chains start to disentangle and align along the direction of the shear force. This results in reduced molecular/particle interactions and increased free space, leading to a decrease in viscosity [54]. It is expected that when subjected to a shearing force, they will change their shape to a more flattened sphere. In a similar context, Ashok et al. [55] reported rheo-small angle light scattering (rheo-SALS) images at different critical shear rates, revealing microstructural changes during shear thinning of a dispersion of poly(3,4-ethylenedioxythiophene)–poly(styrenesulfonate) (PEDOT:PSS), a polyelectrolyte complex (PEC) in water. The 2D pattern changes from circular to elliptical, indicating a more oriented structure as the shearing rate increased.

### 3.2. Flocculation of PEC-CMF and PEC-CNF Systems under Dynamic Conditions

**Figure 4:**
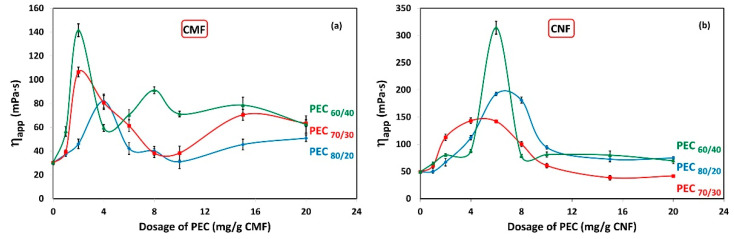
Viscosity as a function of dosage of polyelectrolyte complex (PEC) on (**a**) CMF and (**b**) CNF suspensions. Temperature: 25 °C; concentration: 0.3 wt%; shear rate: 73.38 1/s.

The effect of interactions between PEC and CMF suspensions under dynamic conditions are shown in Figure 4a. It shows the apparent viscosity (*η_app_*) of CMF solutions as a function of PEC dosages. The results indicate a noticeable increase in viscosity at low PEC dosage. The maximum viscosity is achieved at 2 mg PEC/g fiber for PEC_60/40_ and PEC_70/30_, while it reaches 4 mg PEC/g fiber for PEC_80/20_. For PEC_60/40_, an oscillating trend is observed, with a secondary maximum at 8 mg PEC/g CNF. This unexpected behavior can be attributed to the instability of the CMF suspension; however, wall-slip effects cannot be ruled out. Although it is outside the scope of this work, some authors report having found this phenomenon for aqueous dispersions of CMFs [23,56].

Furthermore, Figure 4b illustrates the *η_app_* of CNF solutions in relation to PEC dosages. Surprisingly, the maximum viscosity is observed at 6 mg/g for PEC_60/40_ and PEC_80/20_, and at 4 mg PEC/g fiber for PEC_70/30_. This phenomenon could be attributed to a higher flocculation tendency of CNF suspensions with these specific PEC dosages.

As anticipated, the apparent viscosity of CNFs is higher compared to CMF suspensions.

### 3.3. Flocculation of PEC-CMF and PEC-CNF Systems at Static Conditions

#### Gel Point, Average Floc Size, and ζ-Potential

The gel point represents the critical solids concentration at which flocs form a network by interconnecting with each other. As a result, the minimum gel point corresponds to the maximum floc size. When measuring the gel point in the absence of shear, the process is driven by gravity and Brownian motion. The gel point is determined by plotting the initial solid concentration of CMF and CNF as a function of the following ratio: Final sediment height (h_s_) to the initial (h_0_) height of the suspension. This is shown in Supplementary Information (Figures S2 and S3) considering three different PECs.

**Figure 5:**
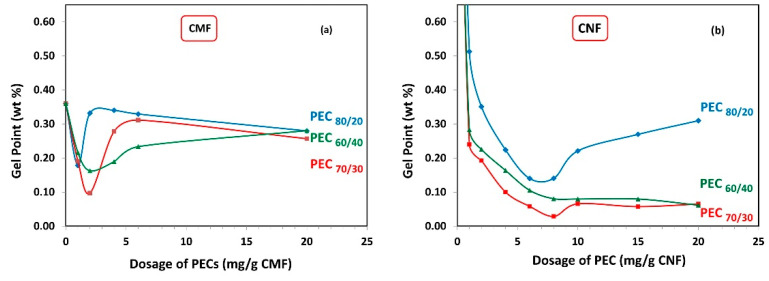
Gel point from sedimentation as a function of dosage of polyelectrolyte complex (PEC) on (**a**) CMF and (**b**) CNF suspensions.

Figure 5a demonstrates the gel point of CMF solutions as a function of PEC dosages. It is observed that with increasing PEC dosage, the gel point flocs reduce in size, reach a minimum, and then start to increase again. The minimum gel point is attained at 2 mg PEC/g fiber for PEC_70/30_ and PEC_60/40_, and 1 mg PEC/g fiber for PEC_80/20_, indicating that low PEC dosage values are required to effectively flocculate the CMF. Furthermore, it is noted that the PEC_70/30_ complex results in the lowest gel point value.

Similarly, Figure 5b shows the gel point of CNF as a function of PEC dosages. The minimum gel point is reached at 8 mg PEC/g fiber for PEC_60/40_ and PEC_70/30_, and 7 mg PEC/g fiber for PEC_80/20_. Notably, there is a distinct difference between the gel point trend between CMF and CNF suspensions, indicating that the formed network has different structures. This finding evidences the important effect of the aspect ratio and electrical charges, which govern the particle–particle interactions induced by PECs. Basically, CMFs have more flexibility due to their larger aspect ratio; however, the electrical charge of them is lower than the electrical charge of CNFs. Consequently, it can be inferred that the CMFs have a better predisposition to entangle in static conditions.

It is important to mention that the final height of sedimentation for the CNF alone cannot be observed, due to the presence of higher repulsive forces that give rise to more stable colloidal suspensions and prevent agglomeration and sedimentation [27]. However, when PEC complexes are added, the gel point data show lower values compared to CMF. For PEC_60/40_ and PEC_70/30_, the gel point decreases, reaches a minimum, and remains at this minimum even as polymer concentration keeps increasing to higher dosages. This indicates that these complexes could be effective flocculants across a wide range of concentrations.

Overall, it is observed that the PEC dosage values where the minimum gel point is achieved are similar for all PECs with different mass ratios, regardless of whether it is for the CMF or CNF suspensions. This suggests that the charge difference of the PECs does not significantly influence this parameter.

**Figure 6:**
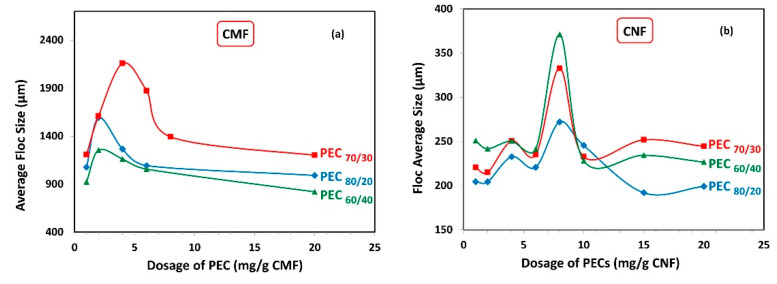
Average floc size as a function of dosage of polyelectrolyte complex (PEC) on (**a**) CMF and (**b**) CNF suspensions.

Figure 6 illustrates the average floc sizes obtained for both the CMF and CNF suspensions. As expected, the average floc size was significantly higher when using CMF compared to CNF, owing to the larger size of the microfibrils.

Interestingly, the maximum floc sizes were observed at the same PEC dosage where the minimum gel points were attained (Figure 5), for both CMF and CNF suspensions. Specifically, in the case of CMF suspensions, the highest floc value was achieved at the PEC_70/30_ ratio, which coincided with the lowest gel point value. Conversely, CNF suspensions demonstrated their smallest average floc size at the PEC_80/20_ ratio, which aligned with the highest gel point value. This behavior can be explained by the fact that more voluminous flocs are less stable and tend to settle due to gravity and their Brownian movements. Consequently, as more PEC is added, smaller but more numerous flocs are produced.

**Figure 7:**
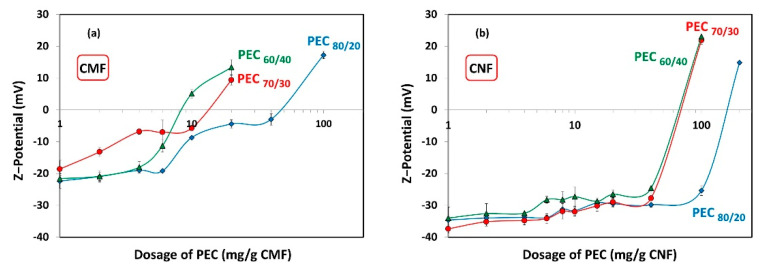
ζ-potential as a function of dosage of polyelectrolyte complex (PEC) on (**a**) CMF and (**b**) CNF suspensions.

Figure 7 shows the ζ-potential results of the different dosages of PECs added to the CMF and CNF suspensions, to assess whether maximum flocculation occurs under anionic, cationic, or neutral conditions. As expected, increasing the cationic charge of the PECs leads to a reduction in the required dosage of PECs to reach the neutrality point of the CMF and CNF suspensions. Specifically, the neutralization of CMF-PEC suspensions was observed at PEC dosages of 8.1 mg PEC/g fiber, 14 mg PEC/g fiber, and 50 mg PEC/g fiber for PEC_60/40_, PEC_70/30_, and PEC_80/20_, respectively. On the other hand, for CNF-PEC, the neutralization occurred at a PEC dosage of 72 mg PEC/g fiber, 74 mg PEC/g fiber, and 165 mg PEC/g fiber for PEC_60/40_, PEC_70/30_, and PEC_80/20_, respectively. This data indicate that the CMFs have a lower charge compared to the CNFs, which translates into an earlier point of neutrality. Additionally, the results highlight that the values of PEC dosages required to achieve neutralization of both suspensions are significantly higher than the necessary dosages to reach the maximum floc; that is, the system is anionic at maximum flocculation.

If the flocculation mechanism was solely based on charge neutralization, the minimum gel point would be at the point of zero charge. However, this is not the case, and the difference can be attributed to two distinct behaviors. Firstly, it is worth considering that CNFs are flexible and smaller than PECs. As a result, the nanofibers may be attracted towards the spherical surface, thereby shielding the positive charges of the complex. Nevertheless, a portion of carboxyl groups of CNFs may remain unneutralized.

**Figure 8:**
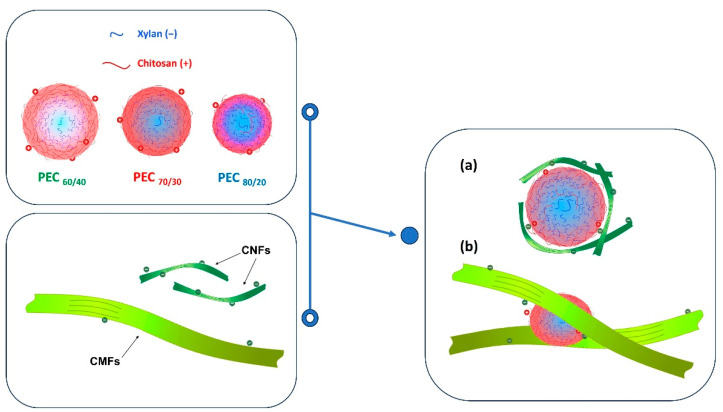
Schematic representation of the interaction of PECs with (**a**) CNFs and (**b**) CMFs.

Secondly, the length of the CMF surpasses the hydrodynamic size of the PEC spheres, allowing the complexes to act as a links, generating a weak network. This weak network formation results from the fact that the surface of the microfibers is not entirely covered by the PEC, and thus, free negative charges persist. Refer to Figure 8 for a visual representation of this scheme.

By considering these two behaviors, the complexities of the flocculation mechanism and its influence on gel point determination can be better understood.

## 4. Conclusions

It has been shown that the Xyl/Ch mass ratio of this PEC clearly defines its characteristics. The greater the amount of xylan added to form the complex, the lower the cationic charge, size, and viscosity of the complex suspension. These PECs produce an effective flocculation of nanocellulose and microcellulose.

Viscosity analysis can detect dynamic flocculation, where the maximum effect doses correlate with those found for static conditions. The maximum flocculation PEC doses were very low and clearly lower than the value required to achieve charge neutralization.

Furthermore, a lower dose of complexes is needed to flocculate CMF than CNF. This reveals the remarkable influence of nanofiber properties, i.e., aspect ratio, charges, and surface area on the flocculation of the system. The results provide valuable information to better understand and analyze the effect of the flocculant action of these PECs on parameters such as the retention of cellulose nano/microfibers and drainage rate in papermaking or the dewatering speed in different industrial systems.

## Figures and Tables

**Table 1:** Characteristics of the micro/nanofibers previously reported by Bastida et al. [27].

	Nanofibrillation Yield (%)	Diameter of CMF Fraction Determined by SEM (nm)	CNF Fraction Determined by TEM		
			Average Diameter (nm)	Average Length (nm)	Average Aspect Ratio (Length/Diameter)
CMF	12 ± 1	190 ± 40	13 ± 4	1200 ± 300	92.3
CNF	54.3 ± 0.3	190 ± 95	12 ± 4	800 ± 200	66.7

**Table 2:** Intrinsic viscosity, ζ-potential, average size, polydispersity (PDI), and critical concentration (c*) of polyelectrolytes solutions (Ch and Xyl) and PECs at different mass ratios.

Sample	Critical Concentration (c*) (wt %)	Intrinsic Viscosity [η] (mL/g)	ζ-Potential (mV) ^(1)^	Average Size (nm) ^(2)^	PDI ^(2)^
Ch	0.04	7610.0	+37 ± 2	---	---
Xyl	>0.6	54.4	−10 ± 2	---	---
PEC_60/40_	0.10	1299.2	+31 ± 1	565 ± 10	0.28 ± 0.03
PEC_70/30_	0.25	690.2	+29.3 ± 0.5	530 ± 5	0.27 ± 0.02
PEC_80/20_	0.30	205.3	+18.3 ± 0.8	400 ± 5	0.23 ± 0.02
CMF	0.03 ^(3)^	367.6 ^(3)^	−27 ± 5 ^(4)^	200 ± 30 ^(4)^	0.61 ± 0.03 ^(4)^
CNF	0.07 ^(3)^	145.5 ^(3)^	−32 ± 4 ^(4)^	80 ± 15 ^(4)^	0.47 ± 0.01 ^(4)^

^(1)^ Values are the average of four replicates. ^(2)^ Values are the average of five replicates. ^(3)^ Values previously reported by [27]. ^(4)^ Values previously reported by [18].

**Table 3:** Rheological parameters (K: consistency index; n: flow behavior index; R^2^: regression coefficient) of solutions of both polyelectrolytes and PECs with different mass ratio (Xyl/Ch: 60/40, 7/30, and 80/20), corresponding to the Power Law model. (0.3 wt% concentration).

Samples	K (1/(mPa·s))	n	R^2^
Xyl	40.3	0.841	0.9731
Ch	181.1	0.915	0.9752
PEC 60/40	89.5	0.808	0.9789
PEC 70/30	77.2	0.779	0.9853
PEC 80/20	43.9	0.841	0.9955

## Data Availability

Not applicable.

## Supplementary Materials

The following supporting information can be downloaded at: https://www.mdpi.com/article/10.3390/nano13172420/s1. Figure S1. Apparent viscosity *(η_app_*) versus shear rate (7.34; 14.68; 36.69 and 73.38 1/s) and different concentration (from 0.1 wt% to 0.6 wt%) for complexes (PECs) at different mass ratio Xyl/Ch: (a) 60/40; (b) 70/30 and (c) 80/20. Temperature: 25 °C. Table S1. Rheological parameters of PECs at different concentration using the Power Law model. Figure S2. Initial solid concentration of CMF vs final sediment height (h_s_)/initial (h_0_) height of suspension. Table S2. Sedimentation data of CMF fitted with a quadratic equation and gel point. Figure S3. Initial solid concentration of CNF vs final sediment height (h_s_)/initial (h_0_) height of suspension. Table S3. Sedimentation data of CNF fitted with a quadratic equation and gel point.
