# Supplementary material for: Flocculation of Cellulose Microfiber and Nanofiber Induced by Chitosan–Xylan Complexes
Source: Nanomaterials (Basel). 2023 Aug 25;13(17):2420. doi: 10.3390/nano13172420 (PMC10489922; doi:10.3390/nano13172420)
Supplement: Supplementary file 1 [file nanomaterials-13-02420-s001.zip › nanomaterials-2563879-supplementary.pdf]

*nanomaterials*

*Article*

# Flocculation of Cellulose Microfiber and Nanofiber Induced by Chitosan-Xylan complexes.

**Gabriela Adriana Bastida [1,2], Quim Tarrés [2], Roberto Aguado [2], Marc Delgado-Aguilar [2], Miguel Ángel Zanuttini [1] and María Verónica Galván [1]**

[1] Institute of Cellulosic Technology, Faculty of Chemical Engineering (FIQ-CONICET), National University of the Litoral, Santiago del Estero 2654, Santa Fe S3000AOJ, Argentina.
[2] LEPAMAP-PRODIS research group, University of Girona. Maria Aurèlia Capmany, 61 – 17003 Girona (Spain)
* Correspondence: ejoaquimagusti.tarres@udg.edu; Tel.:

This file includes Supplementary Figure S1, S2 and S3. Table S1, S2 and S3

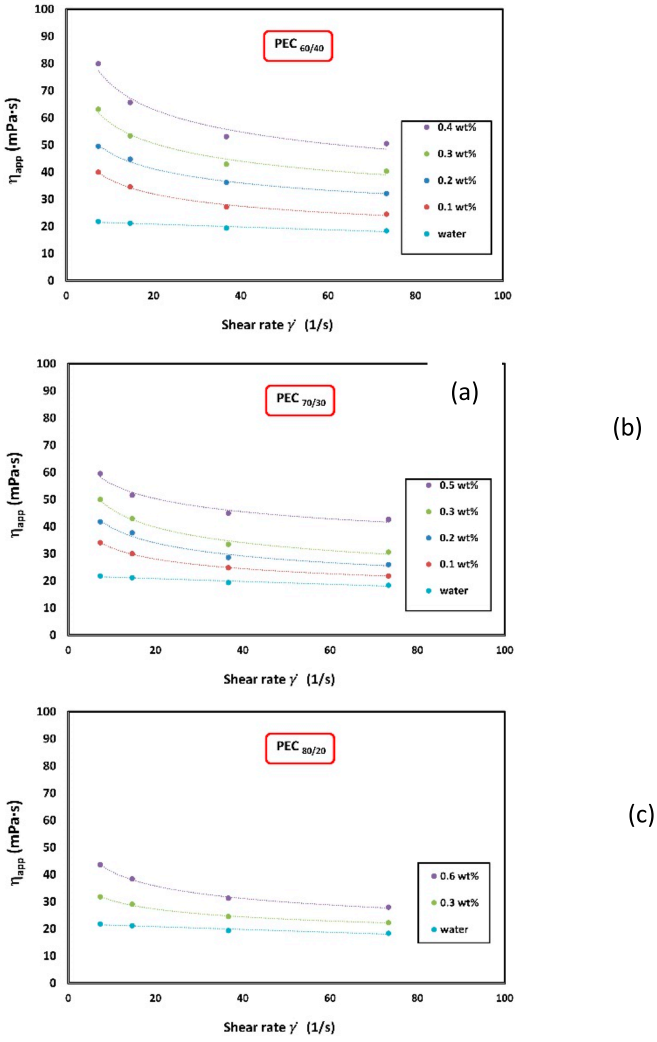


**Figure S1.** Apparent viscosity (ηapp) versus shear rate (7.34; 14.68; 36.69 and 73.38 1/s) and different concentration (from 0.1 wt% to 0.6 wt%) for complexes (PECs) at different mass ratio Xyl/Ch: (**a**) 60/40; (**b**) 70/30 and (**c**) 80/20. Temperature: 25 ºC.

**Citation:** Lastname, F.; Lastname, F.; Lastname, F. Title. *Nanomaterials* **2022**, *12*, x. https://doi.org/10.3390/xxxxx

Academic Editor: Firstname Lastname

Received: date
Accepted: date
Published: date

**Publisher's Note:** MDPI stays neutral with regard to jurisdictional claims in published maps and institutional affiliations.

**Copyright:** © 2022 by the authors. Submitted for possible open access publication under the terms and conditions of the Creative Commons Attribution (CC BY) license (https://creativecommons.org/licenses/by/4.0/).

**Table S1.** Rheological parameters of PECs at different concentration using the Power Law model.

| Samples | Concentration (wt %) | K (1(mPa·s)) | n | $R^2$ |
|---|---|---|---|---|
| PEC $_{60/40}$ | 0.1 | 61.8 | 0.780 | 0.9917 |
| | 0.2 | 73.8 | 0.806 | 0.9933 |
| | 0.3 | 92.4 | 0.799 | 0.9727 |
| | 0.4 | 116.3 | 0.796 | 0.9563 |
| PEC $_{70/30}$ | 0.1 | 50.4 | 0.805 | 0.9995 |
| | 0.2 | 65.5 | 0.781 | 0.9785 |
| | 0.3 | 77.2 | 0.779 | 0.9853 |
| | 0.5 | 77.8 | 0.855 | 0.9679 |
| PEC $_{80/20}$ | 0.3 | 43.9 | 0.841 | 0.9955 |
| | 0.6 | 64.7 | 0.803 | 0.9967 |

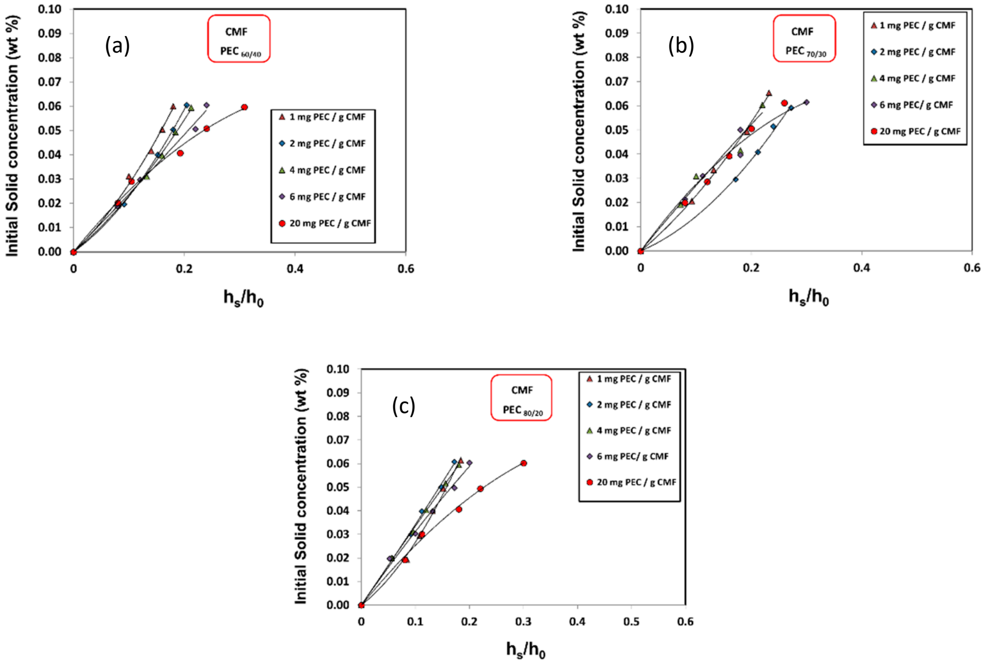


**Figure S2.** Initial solid concentration of CMF vs final sediment height ($h_s$) / initial ($h_0$) height of suspension.

**Table S2.** Sedimentation data of CMF fitted with a quadratic equation and gel point.

| Sample | Dosage of PEC (mg PEC / g CMF) | Y= Ax² + Bx (*) | $R^2$ | Gel Point (wt %) (**) |
|---|---|---|---|---|
| **PEC** $_{60/40}$ | 1 | $Y = 0.6341x^2 + 0.2163x$ | $R^2 = 0.9931$ | 0.2163 |
| | 2 | $Y = 0.6549x^2 + 0.1629x$ | $R^2 = 0.9995$ | 0.1629 |
| | 4 | $Y = 0.4142x^2 + 0.1895x$ | $R^2 = 0.9963$ | 0.1895 |
| | 6 | $Y = 0.0403x^2 + 0.2337x$ | $R^2 = 0.9943$ | 0.2337 |
| | 20 | $Y = -0.2888x^2 + 0.2807x$ | $R^2 = 0.9935$ | 0.2807 |
| **PEC** $_{70/30}$ | 1 | $Y = 0.3785x^2 + 0.1908x$ | $R^2 = 0.9979$ | 0.1908 |
| | 2 | $Y = 0.4561x^2 + 0.0971x$ | $R^2 = 0.9976$ | 0.0971 |
| | 4 | $Y = -0.083x^2 + 0.2783x$ | $R^2 = 0.9711$ | 0.2783 |
| | 6 | $Y = -0.3534x^2 + 0.3115x$ | $R^2 = 0.9767$ | 0.3115 |
| | 20 | $Y = -0.0698x^2 + 0.2570x$ | $R^2 = 0.9976$ | 0.2570 |
| **PEC** $_{80/20}$ | 1 | $Y = 0.8793x^2 + 0.1785x$ | $R^2 = 0.9959$ | 0.1785 |
| | 2 | $Y = 0.0993x^2 + 0.3320x$ | $R^2 = 0.9974$ | 0.3320 |
| | 4 | $Y = -0.0594x^2 + 0.3405x$ | $R^2 = 0.9989$ | 0.3405 |
| | 6 | $Y = -0.1773x^2 + 0.3294x$ | $R^2 = 0.9933$ | 0.3294 |
| | 20 | $Y = -0.2633x^2 + 0.2799x$ | $R^2 = 0.9963$ | 0.2799 |

* Y is initial solid concentration and X is $(h_s)/(h_0)$. ** The first derivate of the curve at the y-intercept gives the gel point.

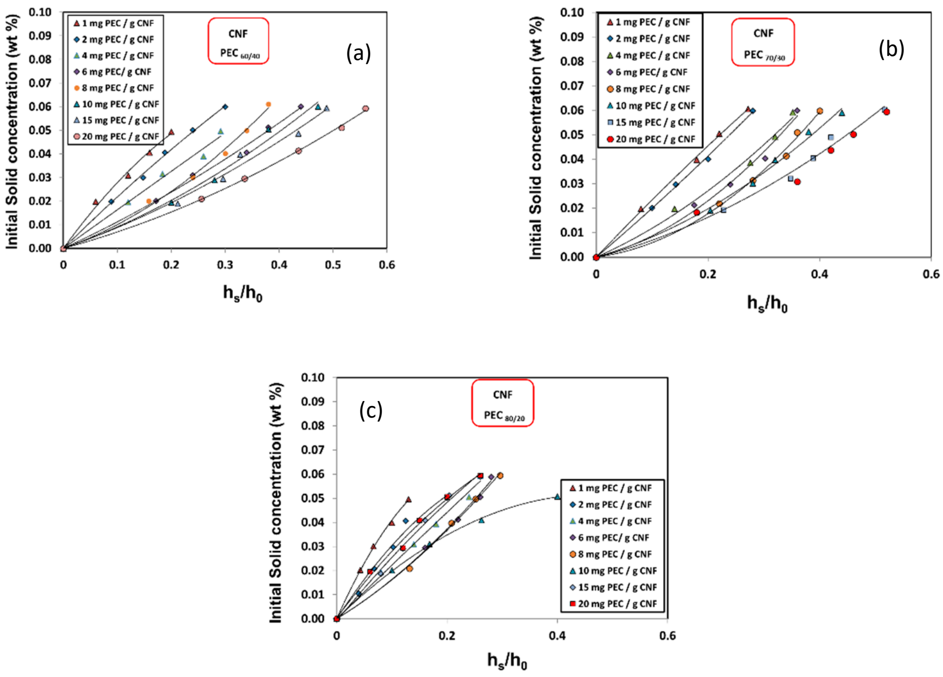


**Figure S3.** Initial solid concentration of CNF vs final sediment height ($h_s$) / initial ($h_0$) height of suspension.

**Table S3.** Sedimentation data of CNF fitted with a quadratic equation and gel point.

| Sample | Dosage of PEC (mg PEC / g CNF) | $Y = Ax^2 + Bx$ (*) | $R^2$ | Gel point (wt %) (**) |
|---|---|---|---|---|
| PEC 60/40 | 1 | $y = -0.3517x^2 + 0.3135x$ | 0.9940 | 0.3135 |
| | 2 | $y = -0.0826x^2 + 0.2258x$ | 0.9981 | 0.2258 |
| | 4 | $y = -0.0022x^2 + 0.1636x$ | 0.9876 | 0.1636 |
| | 6 | $y = 0.1999x^2 + 0.0806x$ | 0.9948 | 0.0806 |
| | 8 | $y = 0.0686x^2 + 0.1054x$ | 0.9938 | 0.1054 |
| | 10 | $y = 0.1094x^2 + 0.0797x$ | 0.9880 | 0.0797 |
| | 15 | $y = 0.0838x^2 + 0.0798x$ | 0.9868 | 0.0798 |
| | 20 | $y = 0.0760x^2 + 0.0618x$ | 0.9992 | 0.0618 |
| PEC 70/30 | 1 | $Y = -0.0447x^2 + 0.2347x$ | 0.9976 | 0.2347 |
| | 2 | $Y = 0.0257x^2 + 0.2043x$ | 0.9959 | 0.2043 |
| | 4 | $Y = 0.1917x^2 + 0.0957x$ | 0.9884 | 0.0957 |
| | 6 | $Y = 0.2601x^2 + 0.0668x$ | 0.9876 | 0.0668 |
| | 8 | $Y = 0.2989x^2 + 0.0289x$ | 0.9942 | 0.0289 |
| | 10 | $Y = 0.1647x^2 + 0.0658x$ | 0.9933 | 0.0658 |
| | 15 | $Y = 0.1199x^2 + 0.0576x$ | 0.9908 | 0.0576 |
| | 20 | $Y = 0.0916x^2 + 0.0651x$ | 0.9860 | 0.0651 |
| PEC 80/20 | 1 | $Y = -1.0331x^2 + 0.5125x$ | 0.9989 | 0.5125 |
| | 2 | $Y = -0.4768x^2 + 0.3511x$ | 0.9837 | 0.3511 |
| | 4 | $Y = -0.0174x^2 + 0.2119x$ | 0.9958 | 0.2119 |
| | 6 | $Y = 0.2303x^2 + 0.1403x$ | 0.9969 | 0.1403 |
| | 8 | $Y = 0.2129x^2 + 0.1408x$ | 0.9980 | 0.1408 |
| | 10 | $Y = -0.2376x^2 + 0.2212x$ | 0.9992 | 0.2212 |
| | 15 | $Y = -0.1450x^2 + 0.2697x$ | 0.9943 | 0.2697 |
| | 20 | $Y = -0.3063x^2 + 0.3099x$ | 0.9923 | 0.3099 |

* Y is initial solid concentration and X is $(h_s)/(h_0)$. ** The first derivate of the curve at the y-intercept gives the gel point.
